# Sb_2_S_3_ grown by ultrasonic spray pyrolysis and its application in a hybrid solar cell

**DOI:** 10.3762/bjnano.7.158

**Published:** 2016-11-10

**Authors:** Erki Kärber, Atanas Katerski, Ilona Oja Acik, Arvo Mere, Valdek Mikli, Malle Krunks

**Affiliations:** 1 Laboratory of Thin Film Chemical Technologies, Department of Materials Science, Tallinn University of Technology, Ehitajate tee 5, 19086 Tallinn, Estonia; 2 Chair of Semiconductor Materials Technology, Department of Materials Science, Tallinn University of Technology, Ehitajate tee 5, 19086 Tallinn, Estonia

**Keywords:** absorber, chemical spray pyrolysis (CSP), hybrid solar cell, stibnite (Sb_2_S_3_), ultrasonic atomization

## Abstract

Chemical spray pyrolysis (CSP) is a fast wet-chemical deposition method in which an aerosol is guided by carrier gas onto a hot substrate where the decomposition of the precursor chemicals occurs. The aerosol is produced using an ultrasonic oscillator in a bath of precursor solution and guided by compressed air. The use of the ultrasonic CSP resulted in the growth of homogeneous and well-adhered layers that consist of submicron crystals of single-phase Sb_2_S_3_ with a bandgap of 1.6 eV if an abundance of sulfur source is present in the precursor solution (SbCl_3_/SC(NH_2_)_2_ = 1:6) sprayed onto the substrate at 250 °C in air. Solar cells with glass-ITO-TiO_2_-Sb_2_S_3_-P3HT-Au structure and an active area of 1 cm^2^ had an open circuit voltage of 630 mV, short circuit current density of 5 mA/cm^2^, a fill factor of 42% and a conversion efficiency of 1.3%. Conversion efficiencies up to 1.9% were obtained from solar cells with smaller areas.

## Introduction

A solution-based technology coupled with earth abundant materials is an attractive path for affordable next-generation solar cells. The present paper relies on the recently proven concept of TiO_2_/Sb_2_S_3_/P3HT solar cells [1–12], in which Sb_2_S_3_ is the light absorber, also called the sensitizer, situated in the electrical junction created by TiO_2_ and P3HT (polythiophene) as the electron and hole conductor, respectively. For these type of solar cells, fluorine doped tin oxide (FTO) is prevalently used to contact the TiO_2_ while evaporated Au has been used to contact the P3HT. The use of a polymeric hole conductor coupled with inorganic materials leads to the notation of a hybrid solar cell [13]. Based on dense TiO_2_ covered by mesoporous TiO_2_ and then impregnated with a light harvester, the resulting solar cells are commonly referred to as mesoscopic solar cells [14] the first of which, based on Sb_2_S_3_ sensitizer, yielded conversion efficiencies above 3% [15–16].

The central part of the particular system, the Sb_2_S_3_ absorber, has so far been produced mainly by chemical bath deposition (CBD) for which the presence of impurities such as antimony hydroxide is inherent, and it is essential to use post-deposition heat-treatment due to the initially amorphous yield [17]. CBD is also considered as not suitable for large-scale applications unless the conventional low-temperature method is replaced by synthesis at room temperature [18]. The solar cells that rely on the CBD-grown Sb_2_S_3_ and meso-porous TiO_2_ as the electron conductor have reached a conversion efficiency of 7.5% when post-deposition sulfurization and thermal treatment of Sb_2_S_3_ were used [17]. The introduction of atomic layer deposition (ALD) for growing Sb_2_S_3_ onto a meso-porous TiO_2_ substrate was successful with respective solar cell efficiencies reaching from 2.6% in the first study in 2013 [19] up to 5.8% in 2014 [5]. The success was attributed to the conformity of the coating by the oxide-free Sb_2_S_3_ layer. Nevertheless, the initial product in ALD is also amorphous and requires an annealing stage to obtain crystalline Sb_2_S_3_.

Another solution-based method, besides CBD, is spin coating, which has been used to obtain Sb_2_S_3_ absorber layers by multiple steps of a coating–annealing procedure [12], or a single-step coating–annealing procedure developed by Choi and Seok in 2015 [20]. The resulting solar cells showed conversion efficiencies of up to 2.3% when based on planar TiO_2_ [12] and 6.4% [20] when based on mesoporous TiO_2_. Spin coating is a simple technique and seems attractive for the deposition of oxide-free Sb_2_S_3_ absorber. However, an annealing stage at 300 °C in inert gas atmosphere is involved. Chemical spray pyrolysis (CSP) is a simple and fast method in which a solution of precursor materials is pulverized and the aerosol is then guided by flow of carrier gas onto a hot substrate. The droplets can be produced pneumatically, or, ultrasonically with a piezoelectric generator submerged in the solution bath. The precursors for Sb and S have been SbCl_3_ and thiourea (tu) [21] or thioacetamide [22], respectively, dissolved in water together with a complexing agent such as tartaric acid to reduce the hydrolysis of the SbCl_3_ in the spray solution [21–22]. We have observed that the use of tartaric acid as the complexing agent results in unwanted residues in the films grown by pneumatic spray [23]. Alternatively, non-aqueous solvents such as acetic acid or alcohols have been utilized to eliminate problems associated with the hydrolysis of SbCl_3_ [22,24]. In general, the aqueous solvent tends to result in amorphous Sb_2_S_3_ films whereas films prepared from non-aqueous solvents have been reported as polycrystalline [22,24]. So far, we have shown that for growing Sb_2_S_3_ by pneumatic CSP the use of SbCl_3_ and thiourea (tu) precursors with an SbCl_3_/tu molar ratio of 1:3 dissolved in methanol and sprayed on substrate at a temperature of 255 °C leads to films that consist of orthorhombic stibnite and a secondary phase that was identified as Sb_2_O_3_ [23]. To suppress the formation of oxides, an excess of sulfur source in the precursor solution may be required as indicated by similar studies for indium sulfide [25–26], zinc sulfide [27] and tin sulfide [28–29] grown by pneumatic CSP. At the time of the present study, no publications were available on ultrasonically spray-grown Sb_2_S_3_, and on the application of any CSP-grown Sb_2_S_3_ in a solar cell, apart from a photoelectrochemical cell that showed an efficiency of 0.3% [30].

In this work, we report the first results on growing Sb_2_S_3_ by ultrasonic chemical spray pyrolysis (ultrasonic CSP), and on the application of ultrasonic CSP grown Sb_2_S_3_ as an absorber in a hybrid solar cell. The aim of this work is to obtain a single-phase Sb_2_S_3_ absorber by ultrasonic CSP, and to test the Sb_2_S_3_ layer in a planar TiO_2_/Sb_2_S_3_/P3HT configuration solar cell. In the present work we will show that by using an excess of thiourea as the sulfur source in the spray solution, such as with a SbCl_3_/tu molar ratio of 1:6, one can rapidly grow single-phase and crystalline Sb_2_S_3_ by ultrasonic CSP without the post-deposition heat treatment stage, yielding solar cell conversion efficiencies up to 1.9% when coupled with a planar TiO_2_ layer, also grown by spray in air.

## Results and Discussion

### Influence of the molar ratio of the precursors on phase composition and morphology of the antimony sulfide layers

**Figure 1:**
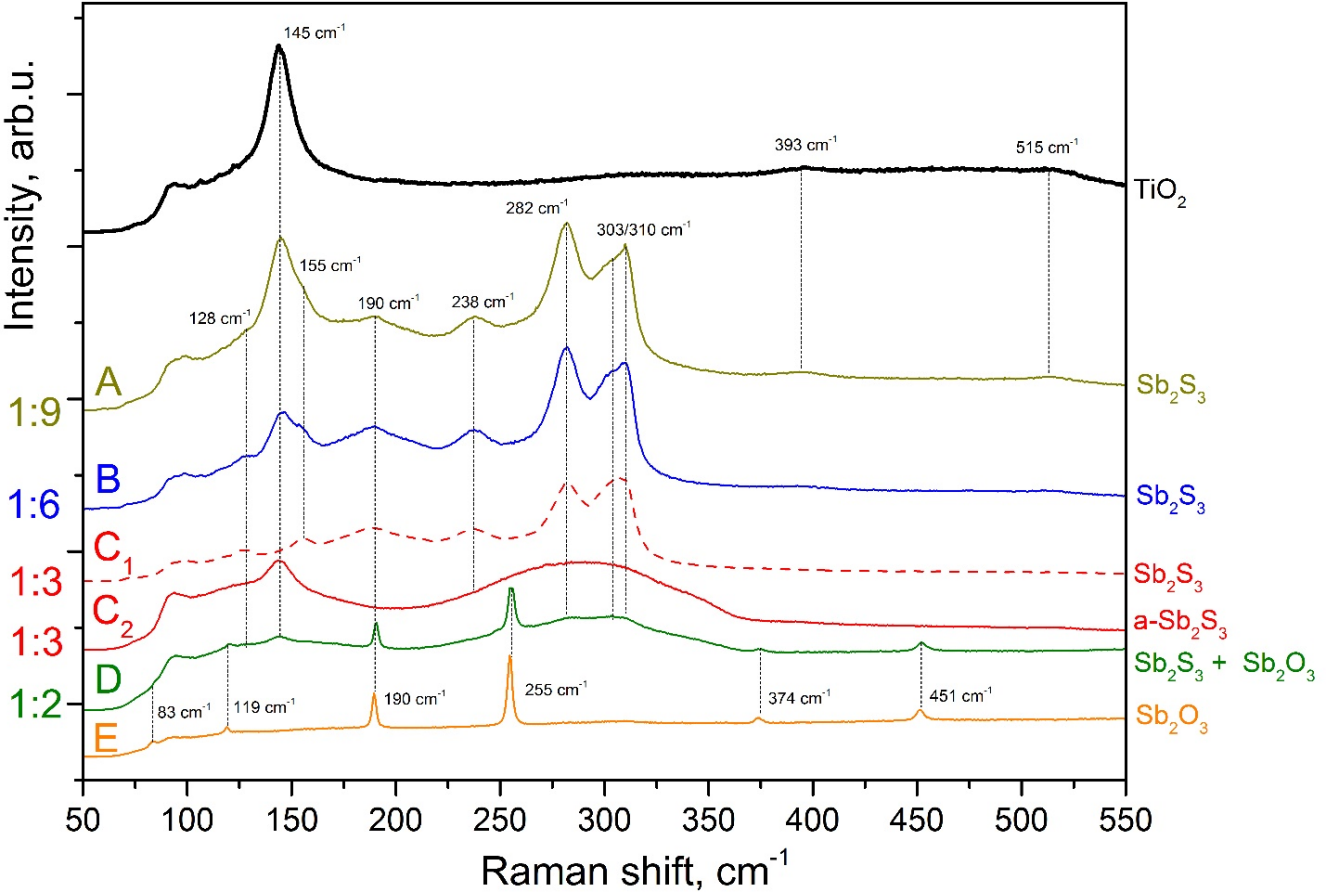
Raman spectra A–D of thin layers grown by spraying solutions with Sb/S precursor ratios of 1:9 to 1:2 onto a glass/ITO/TiO_2_substrate kept at 250 °C. The red dashed line (C_1_) is for spectrum that is collected from areas less common for sample C, the red solid line (C_2_) is for spectrum that is prevailing for sample C. The Raman spectra are measured from glass/ITO/TiO_2_/Sb_2_S_3_ samples after five deposition cycles of Sb_2_S_3_. The spectra remain similar when varying the number of cycles (not shown). As a reference, the Raman spectrum E with peaks attributed to Sb_2_O_3_ belongs to a single layer that was produced by heating the Sb-precursor (SbCl_3_) on a glass substrate at 450 °C in air, and a Raman spectrum of the TiO_2_ substrate layer is also given.

The films obtained by CSP at 250 °C had differed in appearance and homogeneity depending on the molar ratio of the precursors in the solution. The visually homogeneous and dark layers grown from precursor solutions with Sb/S molar ratios of 1:6 and 1:9 on a glass/ITO/TiO_2_ substrate show Raman peaks at 145, 393 and 515 cm^−1^, which were attributed to the anatase phase of TiO_2_ [31], and two prominent peaks at around 282 and 303/310 cm^−1^, which can be attributed to crystalline Sb_2_S_3_ [32–34], see Figure 1, spectrum A and B. Also, the lower intensity peaks at 128 cm^−1^ [32], 155 cm^−1^ [32], 190 cm^−1^ [33] and 238 cm^−1^ [32–34] are expected to belong to crystalline Sb_2_S_3_. Many of these peaks could overlap with those characteristic of various Sb oxides [32–33,35–37]. However, we are guided by the fact that up to 570 °C cubic Sb_2_O_3_ is the most stable form of Sb oxide [38]. We have verified by Raman spectroscopy the peak positions of the cubic Sb_2_O_3_ single layer (Figure 1, spectrum E) produced by heating the Sb precursor (SbCl_3_) on glass at 450 °C in air. The most intense peak of cubic Sb_2_O_3_ is expected to be present at 255 cm^−1^ [39] and is absent in the as-deposited films produced from 1:6 and 1:9 solutions, suggesting that the oxidation of the Sb precursor is negligible when an abundance of sulfur source is provided in the solution.

**Figure 2:**
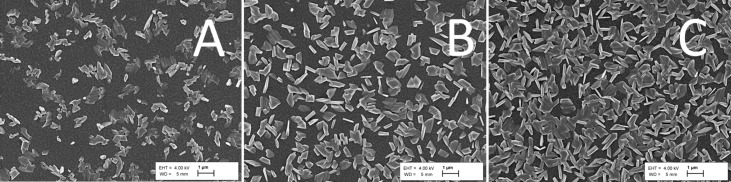
SEM images of Sb_2_S_3_ crystalline flakes grown by spraying solutions with a precursor ratio of 1:6 onto a TiO_2_ substrate kept at 250 °C using 3, 7 and 11 deposition cycles, depicted in images A, B and C, respectively. The linear dimensions of the flakes, or coalesced flakes, are in the range of 200–800 nm, 500–1200 nm and 800–1500 nm in panels A, B and C, respectively.

On the contrary, the layers grown from 1:3 solutions are heterogeneous. The 1:3 layers show prevailing regions that are visually transparent with an orange hue, and more sparsely located dark regions. Also the surface image by SEM (Supporting Information File 1, Figure S1a) shows varying rough areas with grains and smoother areas with no grains in the layer. The Raman spectra of the layer produced from 1:3 solution is presented in Figure 1 as spectrum C_1_ (dashed line, characteristic of the dark regions), and as spectrum C_2_ (solid line, characteristic of the orange colored regions). The broad band centered around 290 cm^−1^ in spectrum C_2_ is attributed to amorphous antimony sulfide as in other antimony sulfide layers produced by CBD [7,16,40–41] and by ALD [5,19]. The peak at 145 cm^−1^ attributed to TiO_2_ is also more pronounced in spectrum C_2_, presumably due to the higher transparency of the amorphous layer to the green laser beam when compared to regions with higher crystallinity (spectrum C_1_). Secondly, the 145 cm^−1^ peak appears to be more intense as we increase the precursor ratio from 1:3 to 1:6 (spectrum C_1_ vs B), or increasing from 1:6 to 1:9 (spectrum B vs A). As the increase in thiourea content in the spray solution leads to layers composed of particles with gaps in between that leave the TiO_2_ substrate partly exposed (SEM images for 1:6 samples to be discussed below are presented in Figure 2), it seems reasonable to assume that the coverage of TiO_2_ by Sb_2_S_3_ dictates the emerging of the Raman signal at 145 cm^−1^, which was attributed to the TiO_2_ substrate.

To further scrutinize the phase composition of the layers, and to verify the effect of the precursor ratio, we have presented Raman spectra of layers grown from 1:2 solution. When evaluated by Raman spectroscopy, the 1:2 layer was very heterogeneous. To illustrate this we have chosen one spectrum that clearly shows the signal from antimony oxide (Figure 1, spectrum D). The presence of oxide is indicated by the sharp peaks at 83 cm^−1^ [36–37], 119 cm^−1^ [35,37], 190 cm^-1^ [32–33,35,37,39,42–43], 255 cm^−1^ [32–33,35,37,39], 374 cm^−1^ [32–33,35,39] and 452 cm^−1^ [32–33,35,37,39] that correspond to cubic Sb_2_O_3_. According to the SEM study, sparsely distributed pyramidal crystals were present in the layer (Supporting Information File 1, Figure S1b) that according to EDX were composed of antimony and oxygen only. Such Sb_2_O_3_ crystals have also been reported to be present after annealing the CBD-grown antimony sulfide films at 300 °C [44]. Indeed, the lowering of the content of the sulfur source in the spray solution is expected to favor the formation of oxide phases when growing metal sulfide layers by spray pyrolysis [25–29]. Additionally, we note that the amorphous regions in the layer produced from 1:3 solutions (Figure 1, spectrum C_2_) after annealing at 320 °C for 30 min in N_2_ atmosphere showed the presence of crystalline Sb_2_O_3_ in addition to crystalline Sb_2_S_3_ when evaluated by micro-Raman spectroscopy (not shown), indicating the presence of a non-crystalline oxygen-containing phase prior to annealing in N_2_. EDX results support this proposition as the S/Sb atomic ratio of 1.3 indicates a deficiency of sulfur (when compared to the expected S/Sb ratio of 1.5 in the Sb_2_S_3_ target compound) in the layers as-grown with a precursor ratio of 1:3. Thus, the use of Sb/S precursor ratios higher than 1:3 in the spray solution clearly has an advantage when aspiring an oxide-free Sb_2_S_3_ absorber layer.

In summary, based on Raman spectroscopy, oxide-free layers of crystalline Sb_2_S_3_ can be grown by spray pyrolysis onto the TiO_2_ substrate at 250 °C in air by using precursor ratio of 1:6 or 1:9 in the solution, yielding the use of 1:6 ratio as sufficient. Following this result we will regard 1:6 ratio as an optimum to produce Sb_2_S_3_ absorber layers for the TiO_2_/absorber/P3HT solar cells.

### Evolution of the morphology and optical properties of Sb_2_S_3_ layers with a varying number of deposition cycles

According to the SEM study, the layers grown from 1:6 solutions consist of flake-like particles, and coalesced flakes, with gaps in between that leave the TiO_2_ substrate partly exposed (Figure 2). The flakes are composed of S and Sb with a ratio of 1.53 measured by EDX that within measurement uncertainty corresponds to stoichiometric Sb_2_S_3_.

The number density of Sb_2_S_3_ flakes on the TiO_2_ substrate increases with the number of growth cycles, suggesting that some of the nucleation sites are preferential. The nucleation of secondary Sb_2_S_3_ crystals is evident when using a higher SEM magnification (Supporting Information File 1, Figure S2). In addition, the flakes grow laterally and in height, the approximate sizes of the flakes are presented in the caption of Figure 2. The resulting increase of the overall coverage density is in correspondence with the decrease of the optical transmittance (Supporting Information File 1, Figure S3).

**Figure 3:**
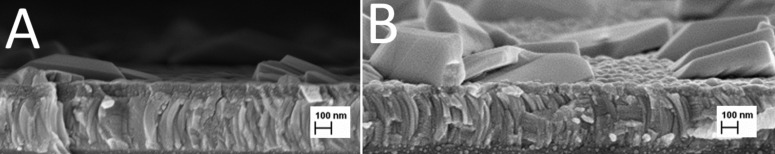
Cross-sectional SEM image of the glass/ITO/TiO_2_/Sb_2_S_3_ structure, the topmost Sb_2_S_3_ layer consists of nanoparticles grown by ultrasonic CSP using three spray cycles (A) or nine spray cycles (B).

For the calculation of the absorption coefficient α, we used 100 nm as a rough estimation of the optically effective thickness of the Sb_2_S_3_ layer based on the SEM image of Sb_2_S_3_ grown using three cycles (Figure 3).

**Figure 4:**
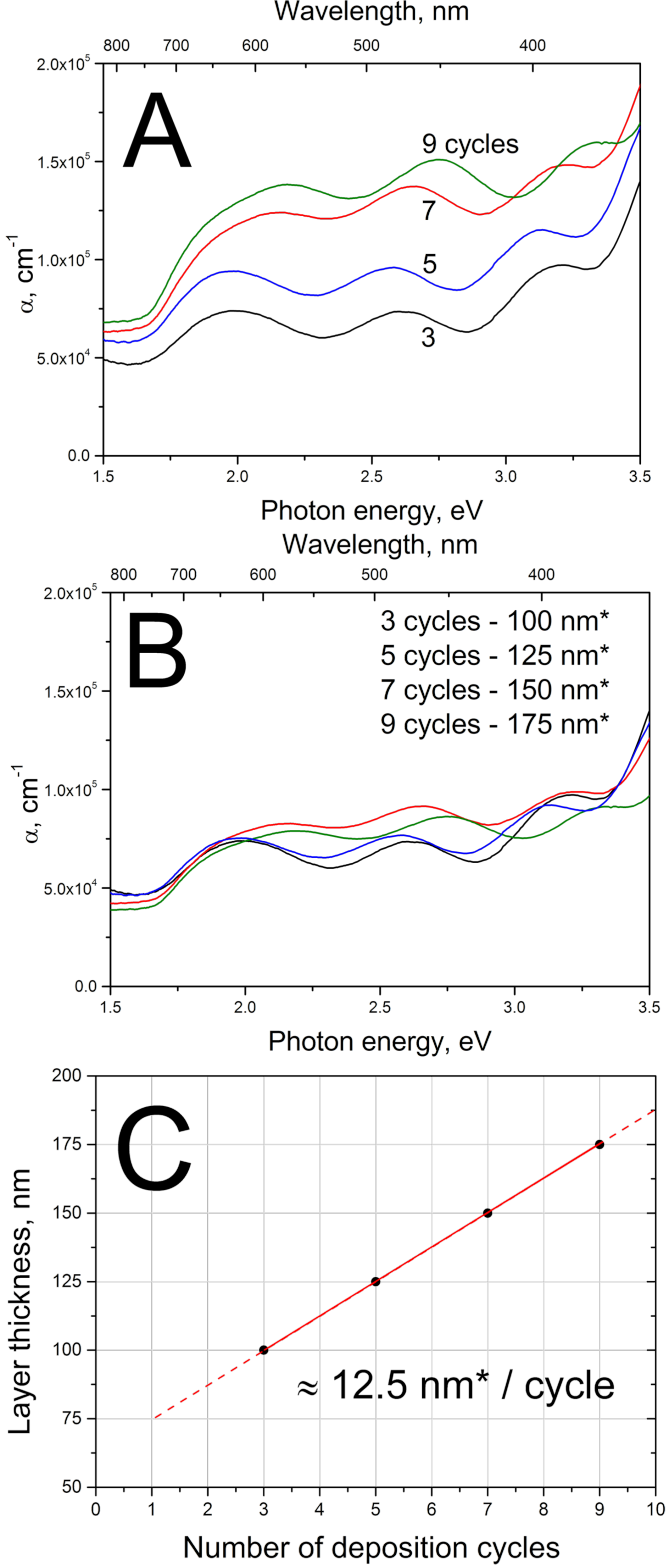
A: Absorption coefficient α of Sb_2_S_3_ layers of nanoparticles grown by ultrasonic CSP with 3–9 deposition cycles, calculated based on the optical transmittance of the glass/ITO/TiO_2_/Sb_2_S_3_ stack, assuming a Sb_2_S_3_ layer thickness of 100 nm; B: α, after applying a correction for the effective thickness by fitting, the units with the asterisk (nm*) stand for the fitted optical thickness; C: growth rate of the Sb_2_S_3_ layer based on the fitted effective layer thicknesses. The interference in each spectrum is due to the transparent thin film ITO/TiO_2_ substrate; the bandgap of TiO_2_ is around 3.3 eV.

The resulting values of the absorption coefficient α of the Sb_2_S_3_ layers are presented in Figure 4. The α values remain above 5 × 10^4^ cm^−1^ right from the absorption edge at 1.6 eV, throughout the visible region 1.8–3.1 eV, and in the near-UV region (Figure 4A). The high values of α are in accordance with those in literature in which we find α values of (7–9) × 10^4^ cm^−1^ in the range of 2.0–3.5 eV for crystalline Sb_2_S_3_ with an *E*_g_ of 1.7 eV prepared by RF sputtering [45]. The absorption coefficients of the layers grown with more than three cycles are likely to be overestimated (Figure 4A) due to the similar thickness of 100 nm assumed for all layers of Sb_2_S_3_ nanoparticles. Thus, a correction for the effective layer thicknesses was made by fitting with the criterion of overlapping values of α for all samples (Figure 4B). For example, the estimation of 175 nm for the effective optical thickness of the nine cycles of Sb_2_S_3_ sample seems to be apt, since the height of the flakes in the nine cycles of Sb_2_S_3_ sample remains at 200–300 nm (Figure 3B).

However, we are herein not so much interested in the absolute values of the thicknesses of the layers, considering that the obtained optical thicknesses are not expected to exactly coincide with the height of the nanoparticulate layers seen from SEM images. Also, in addition to the effective layer height, the effective optical thickness will be influenced by the increase of the coverage as was seen in Figure 2. Instead, the growth rate of the spray-grown Sb_2_S_3_ is more informative, and can be plotted (Figure 4C) based on the fitted effective optical thicknesses.

Considering that a cycle lasted for 3 min, the deposition rate of 12.5 nm per cycle accounts to 0.07 nm·s^−1^ for the ultrasonic spray method, in which we use raw chemicals for the deposition of crystalline Sb_2_S_3_ at around 250 °C in air. A higher but close to comparable growth rate of 0.14 nm·s^−1^ is reported for growing Sb_2_S_3_ by RF sputtering from a preformed high-purity Sb_2_S_3_ target, in vacuum environment, and with a post-deposition annealing at 400 °C under sulfur vapor to obtain crystalline, dense, smooth and stoichiometric Sb_2_S_3_ films [45]. For ALD, the growth rate of Sb_2_S_3_ film has been reported to be 0.002 nm·s^−1^ (0.056 nm·cycle^−1^, 33.5 s·cycle^−1^) prior to annealing in H_2_S atmosphere [5]. Similarly, the conventional CBD requires hours of processing time prior to the annealing procedure [46–47], yielding growth speeds around 0.003 nm·s^−1^ for Sb_2_S_3_ films [47].

**Figure 5:**
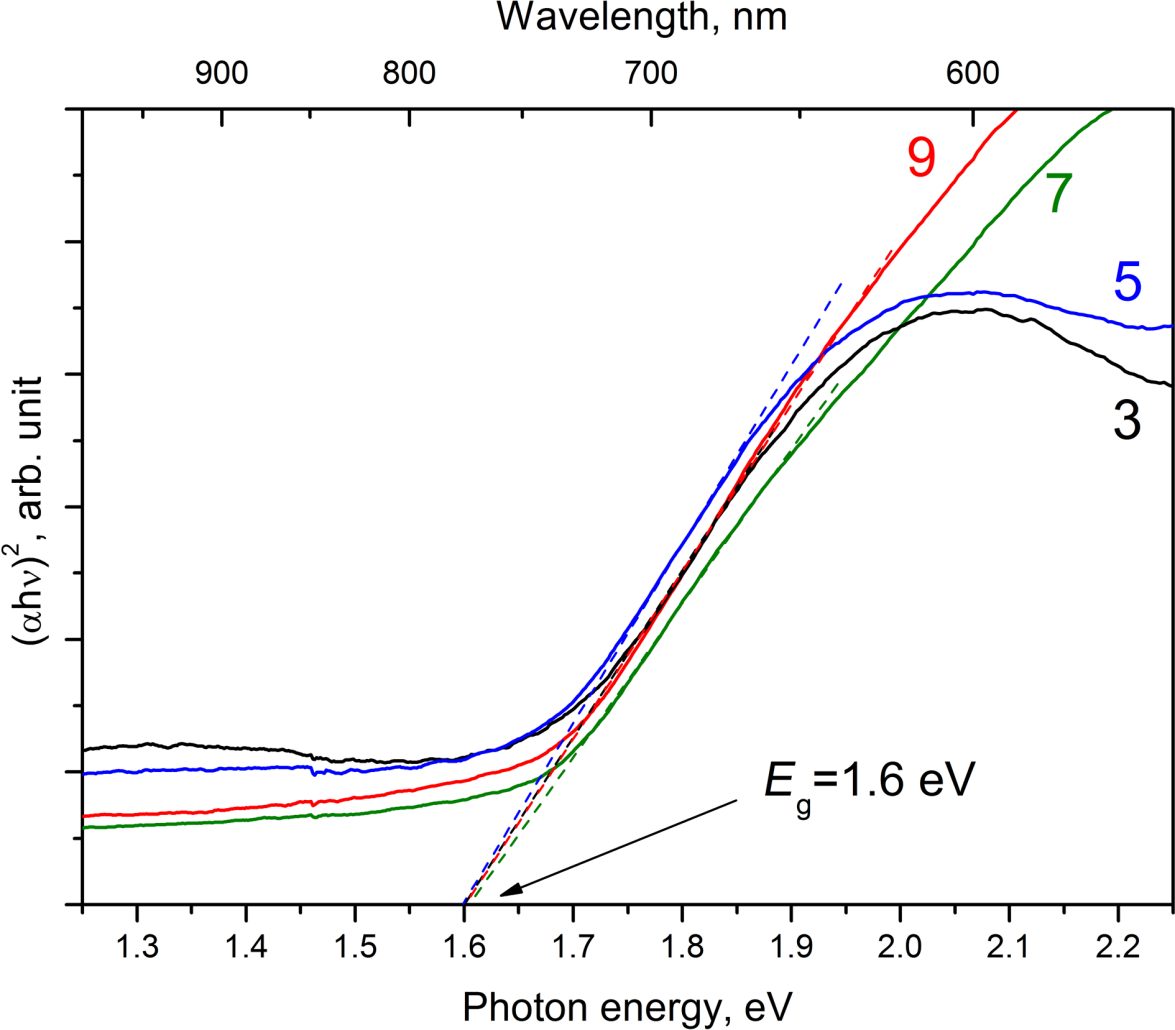
Tauc plot of the optical transmittance spectra of the glass/ITO/TiO_2_/Sb_2_S_3_ layer stack, using α from Figure 4B. The linear part of the absorption edge when extended to the *x*-axis at *y* = 0 is used to determine the bandgap of Sb_2_S_3_. Sb_2_S_3_ was grown for three, five, seven and nine deposition cycles by exposing a glass/ITO/TiO_2_ substrate kept at 250 °C to a spray solution containing SbCl_3_ and SC(NH_2_)_2_ at a molar ratio of 1:6.

The bandgap (*E*_g_) of Sb_2_S_3_ was 1.6 eV irrespective of the number of deposition cycles used when grown using spray of 1:6 solutions onto a glass/ITO/TiO_2_ stack (Figure 5). The bandgap of the Sb_2_S_3_ was calculated using the spectra of the absorption coefficient of the glass/ITO/TiO_2_/Sb_2_S_3_ layer stacks (Figure 4B). The bandgaps obtained are in accordance with an *E*_g_ of 1.55–1.72 eV reported for Sb_2_S_3_ films deposited by spray pyrolysis [48]. Theoretical calculations predict an even lower direct optical transition of 1.40 eV [49]. An absorber bandgap of 1.65 eV is found in solar cells with Sb_2_S_3_ prepared by ALD [5,19] or in solar cells with Sb_2_S_3_ prepared by CBD, as estimated from the photocurrent edge at around 750 nm in the published EQE plots [2,4,6–9,11–12,15–17,40–41,50–53]. Any *E*_g_ larger than 1.7 eV up to 2.6 eV have been attributed to nanocrystalline Sb_2_S_3_ [1,44,54], or to amorphous Sb_2_S_3_ [6,44–45,53], while it is also known that contamination, most notably with oxygen, can significantly increase the bandgap value of metal sulfide films [23,55–56]. Values of 1.52–1.55 eV are reported for layers of nanotubes, -rods, or -flakes that consist of single phase Sb_2_S_3_ [57–59]. A study on the correlation between the Sb/S ratio in the films and the corresponding values of *E*_g_ suggest that values around 1.6 eV are expected for crystalline and stoichiometric Sb_2_S_3_ (S/Sb = 1.5) films [60]. Thus, based on the low optical bandgap of 1.6 eV, along with the S/Sb ratio of 1.53 obtained by EDX, we can deduce that layers of single-phase stoichiometric Sb_2_S_3_ crystals can be prepared by using the CSP technique.

To sum up, based on the single-phase Sb_2_S_3_ composition of the layers as determined by Raman spectroscopy, and supported by the optical study, namely a bandgap of 1.6 eV, as well as an absorption coefficient of ca. 10^5^ cm^−1^ in a wide region of optical photon energies, and the high growth rate of 0.07 nm·s^−1^ as evaluated by using optical transmittance spectroscopy, we can conclude that Sb_2_S_3_ grown by ultrasonic CSP can be considered as a candidate for the use as an absorber material in a solar cell based on an inorganic sensitizer. We emphasize that the end product of a single-stage growth at 250 °C in air was crystalline and oxide-free Sb_2_S_3_. No additional annealing was needed. This alone can be considered an advantage when comparing the potential of spray to other solution based methods, such as CBD or spin coating, for growing Sb_2_S_3_.

### Properties of the TiO_2_/Sb_2_S_3_ nanoparticle/P3HT solar cell

The highest efficiencies of each cell were observed after being exposed to AM1.5 illumination for up to 45 min, varying from sample to sample. The general trends in the evolution of the current–voltage curve and series resistance, due to light soaking, are presented in the Supporting Information File 1 (Figure S4). The study of the cause behind this light-soaking effect is not within the scope of the present work. Proposed explanations for this behavior include the filling of electron traps in TiO_2_ near the TiO_2_/P3HT interface [61], adsorbed oxygen released during light soaking from TiO_2_ and photocatalytic effects due to the presence of TiO_2_ [40,52,61].

**Figure 6:**
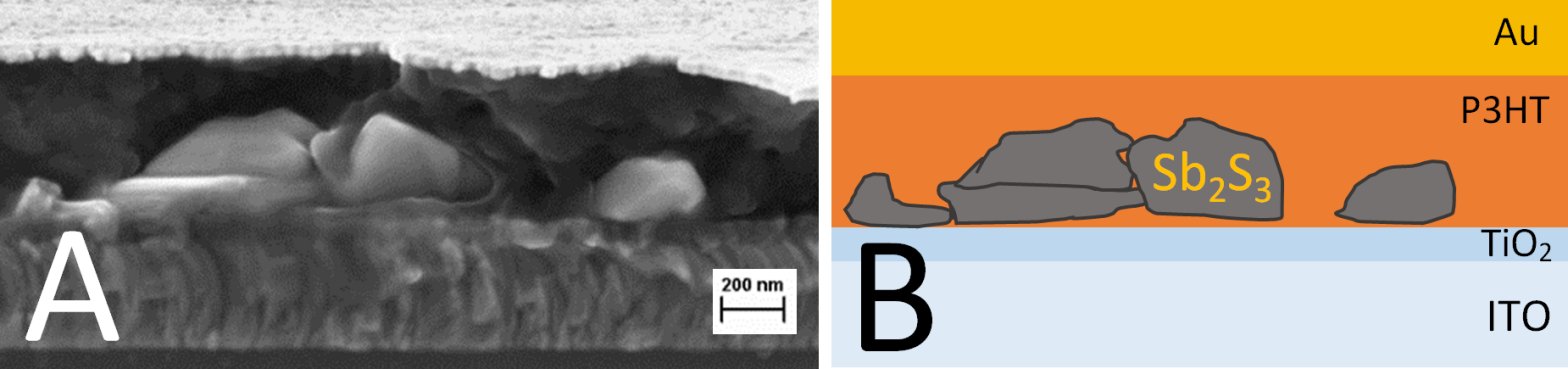
A: Cross-sectional SEM image of the glass/ITO/TiO_2_/Sb_2_S_3_/P3HT/Au solar cell, the 5-cycle Sb_2_S_3_ layer consists of nanoparticles grown by ultrasonic CSP, and B: a corresponding sketch of the solar cell.

An SEM image of the cross-section of the glass/ITO/TiO_2_/Sb_2_S_3_ structure, of the glass/ITO/TiO_2_/Sb_2_S_3_/P3HT/Au structure, and a sketch of the latter, is presented in Figure 6.

### Current–voltage (*I*–*V*) parameters

The principal characteristics of record solar cells with a spray-grown Sb_2_S_3_ absorber prepared using Sb/S of 1:6 in the precursor solution and seven spray cycles are presented in the first two rows of Table 1. The *V*_OC_ of about 618 mV is comparable to that obtained in a similar planar device that uses Sb_2_S_3_ prepared by spin coating [12] and lower than that obtained in a cell that uses mesoporous TiO_2_ and P3HT [3]. The current density of 6 mA·cm^−2^ is on the low side when compared to values above 10 mA·cm^−2^ obtained in devices that rely on the mesoporous TiO_2_ as the electron conductor, or when compared to a planar cell based on ALD-grown Sb_2_S_3_ [5]. The latter corroborates the benefit of a conformal coating and controlled thickness that is characteristic for ALD. As we have previously shown, the use of ZnO nanorods as the structured substrate and an electron conductor for a spray-grown absorber will provide a gain of the current density up to 2.5 times when compared to a planar device [62–63]. Also, the gap filling by a polymeric hole conductor could be expected to be easier in case vertical nanorods are used instead of porous TiO_2_ [13,64–65], current densities as high as 17 mA·cm^−2^ has been obtained using vertical nanowires of TiO_2_ [7]. The search for higher fill factors correlates with the search for the best hole-conductor [51], the highest fill factors have been obtained by using PCPDTBT as the hole conductor (Table 1).

**Table 1:** A review of the main parameters (*V*_OC_, *J*_SC_, FF, eff.) of solid state solar cells that use Sb_2_S_3_ absorber on top of a planar, fibrous, or mesoporous (mp) TiO_2_ layer. The technologies for growing Sb_2_S_3_ are denoted as “spray” for chemical spray pyrolysis, CBD for chemical bath deposition, ALD for atomic layer deposition, and “spin-c.” for spin coating.

TiO_2_ morphology	Sb_2_S_3_ technology	hole conductor(s)	*V*_OC_, mV	*J*_SC_, mA/cm^2^	FF, %	eff., %	area, cm^2^	year	ref.

planar	spray	P3HT	618	6	51	1.9	0.017	2016	this study
planar	spray	P3HT	635	5	42	1.3	1	2016	this study
planar	spin-c.	P3HT	616	8.1	46	2.3	0.16	2015	[12]
mp	spin-c.	PCPDTBT	596	16	67	6.4	0.12	2015	[20]
mp	CBD	PCPDTBT–PCBM and PEDOT:PSS	548	14	68	5.1	n.a.^a^	2015	[18]
mp	spin-c.	P3HT	680	9.5	52	3.4	n.a.^a^	2014	[3]
planar	ALD	P3HT and PEDOT:PSS	666	15	58	5.8	0.16	2014	[5]
mp	CBD	PCPDTBT	711	16	65	7.5	0.16	2014	[17]
mp	CBD	P3HT and PEDOT:PSS	550	13	62	4.4	0.12	2014	[4]
planar	CBD	CuSCN	455	12	59	3.3	0.1	2013	[50]
planar	CBD	P3HT	630	6.1	35	1.4	0.09	2013	[10]
mp	CBD	polyaniline nanobelts	1100	6.9	50	3.8	0.12	2013	[66]
nanofiber	CBD	P3HT and PEDOT:PSS	603	9.9	39	2.3	0.04	2013	[11]
nanowire	CBD	P3HT and PEDOT:PSS	500	17	53	4.5	0.31	2012	[7]
mp	CBD	PCPDTBT-PCBM	595	16	66	6.3	0.16	2012	[9]
mp	CBD	P3HT:Au	626	13	61	4.9	0.16	2012	[2]
mp	CBD	CuSCN	584	13	53	4.1	0.25	2012	[53]
mp	CBD	PCPDTBT	616	15	66	6.2	0.1	2011	[51]
mp	CBD	spiro-MeOTAD	610	11	48	3.1	0.49	2010	[16]
mp	CBD	CuSCN	560	12	58	3.7	0.54	2010	[52]
mp	CBD	CuSCN	490	14	49	3.4	0.15	2009	[15]

^a^Not available, has not been disclosed in the paper.

It is known that a higher active area of the solar cells tend to result in lower FF and lower conversion efficiency, attributed to resistance losses [15]. Nevertheless, larger areas are preferred in the long term. In the second row of Table 1, we have presented cell parameters from 1 cm^2^ area, reflecting the results on the largest area declared so far for TiO_2_/Sb_2_S_3_/P3HT-type solar cells. Indeed, a loss is present when compared to results from the 0.017 cm^2^ area solar cell, the FF decreases from 51% to 42%, and the conversion efficiency decreases from 1.9% to 1.3% brought about by an increase in the series resistance from 2.7 Ω cm^2^ to 30 Ω cm^2^.

The results reflect that we have reached a decent entry for the studies of Sb_2_S_3_-based solar cells and further development looks very promising, e.g. when varying the hole conductor, or, changing the morphology of the layer responsible for the collection of electrons from planar to nanostructured. In either configuration, flat or structured, the Sb_2_S_3_ is expected to be a continuous absorber layer. Such a further development, a conformal Sb_2_S_3_ layer deposited by ultrasonic spray on top of a nanostructured electron conducting substrate, is presently in progress.

### External quantum efficiency

**Figure 7:**
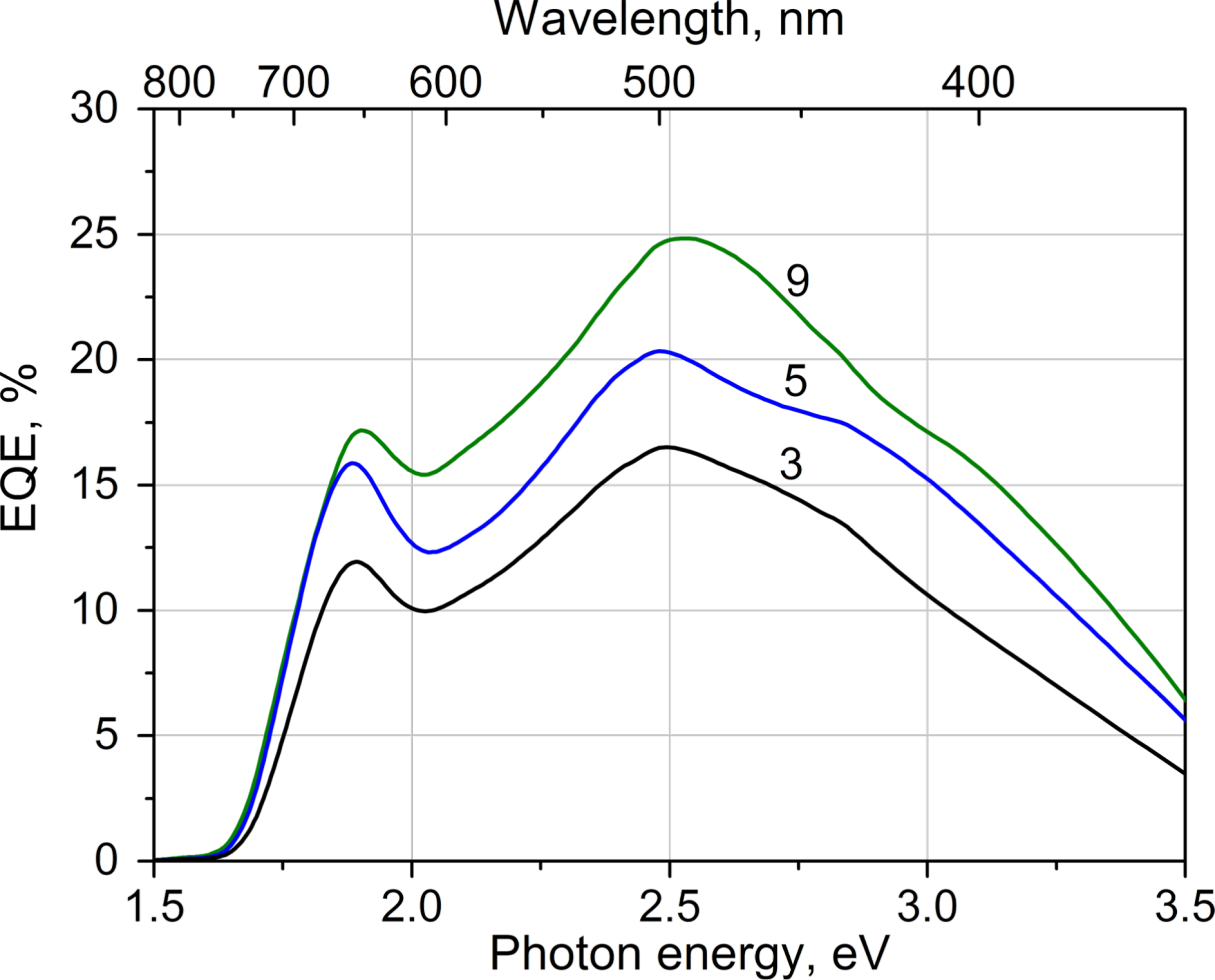
External quantum efficiency of glass/ITO/TiO_2_/Sb_2_S_3_/P3HT/Au solar cells. Sb_2_S_3_ layer was grown by spray pyrolysis of a precursor solution with an Sb/S molar ratio of 1:6 using three, five and nine deposition cycles.

The external quantum efficiency (EQE) of the glass/ITO/TiO_2_/Sb_2_S_3_/P3HT/Au solar cell is presented in Figure 7. The gain of EQE is almost linear when increasing the number of cycles from three to nine, which could be expected considering a homogeneous decrease of the transmittance of the glass/ITO/TiO_2_/Sb_2_S_3_ stack (Supporting Information File 1, Figure S3). The photocurrent edge at around 1.6 eV (775 nm) corresponds well to the absorption edge of the ITO/TiO_2_/Sb_2_S_3_ stack (Figure 5).

The absorption by P3HT has an onset at 1.9 eV (650 nm) [3,8], and it can be seen that there is a depression of EQE in the absorbing region of P3HT (Figure 7). This was also the observation in the case of a cell based on mesoporous TiO_2_ but with otherwise similar stack order, the decline was not observed in the case of other hole conductors used such as PCPDTBT [51]. The conclusion is that the photogenerated carriers of different type in the Sb_2_S_3_ absorber should ideally be separated into their respective conductor layers. However, the additional generation of electron–hole pairs in P3HT adversely affects the carrier separation. This filter effect due to absorption in P3HT was overcome by the formation of PCBM electron channels that bridge the hole conductor directly to TiO_2_ [9,18]. Thus, even for a planar cell, we can expect higher current densities than the 6 mA·cm^−2^ obtained (Table 1) when alternative hole conductors will be exploited.

The short-circuit current *J*_SC_ and the accompanied increase of the conversion efficiency of the glass/ITO/TiO_2_/Sb_2_S_3_/P3HT/Au solar cells had their maximum at seven deposition cycles of Sb_2_S_3_, and the results are presented in Table 1. When adding growth cycles, the initial rise in the conversion efficiency is due to the rise in *J*_SC_ owing to the added volume of Sb_2_S_3_ absorber layer, followed by a decline after seven cycles of Sb_2_S_3_ deposition owing to the drop of FF of the cell (not shown). Such behavior reflects the inevitable need to optimize absorber thickness. The observed decline in the solar cell performance after the optimum absorber thickness is reached has been attributed to losses due to increased recombination in thicker absorber layers [8,10–12]*.* The optimum thickness herein was reached at seven cycles of Sb_2_S_3_ grown by CSP, which corresponds to a fitted Sb_2_S_3_ thickness of 150 nm (Figure 4B). For comparison, for a planar cell with CBD grown Sb_2_S_3_ the optimum Sb_2_S_3_ thickness was found to be 250 nm [10].

By integrating the EQE spectra of a cell over the A.M1.5 solar spectrum, one can evaluate the current density that a particular cell can ideally produce under standard irradiance. Such an integration product should ideally be comparable to the *J*_SC_ obtained from the *I*–*V* measurements. For the EQE presented in Figure 7, the integration products for the samples with three, five and nine deposition cycles are 4.1, 5.5 and 6.4 mA/cm^2^, respectively. This sequence compares fairly well to that obtained from the *I*–*V* measurements: 2.7, 4.4 and 5.7 mA/cm^2^, respectively. The variance is not alarming at this point of studies and can, in the first approach, be attributed to differences in the measurement conditions. Namely, the EQE measurements were performed under low-intensity monochromatic light to avoid any effects owing to the exposure to white light, as opposed to the *I*–*V* measurements performed under the A.M1.5 conditions. The photoconductivity of the hole conductor, as discussed above, might also contribute to the variance between the integrated EQE and the *J*_SC_. Table 1 also presents the *J*_SC_ of 6 mA·cm^−2^ for a cell that had seven cycles of Sb_2_S_3_ deposited, well in line with the *J*_SC_ values for small area samples with five and nine cycles of Sb_2_S_3_ deposited. Ultimately, the true focus of the present study was to obtain a single phase absorber material by spray technique, and the preliminary test of the performance of the material in a solar cell. Optimization of the current density and spectral response is our next goal.

To sum up, at the moment the major reasons behind the low current densities are likely the lack of a continuous Sb_2_S_3_ layer, and/or a non-optimal choice of the hole conductor, as also emphasized in the previous section. To further boost the current density, one is likely to benefit from the use of a stuctured electron-conducting substrate such as a layer of ZnO nanorods.

## Conclusion

We focused on the optimization of the properties of Sb_2_S_3_ grown by chemical spray pyrolysis (CSP) of an ultrasonically nebulized precursor solution, and on the application of ultrasonic CSP grown Sb_2_S_3_ as an absorber in a hybrid solar cell.

The use of ultrasonic CSP resulted in the growth of layers that consist of sub-micrometer single-phase crystalline Sb_2_S_3_ particulates that cover the TiO_2_ substrate homogeneously in case an abundance of the sulfur source (Sb/S molar ratio 1:6 or above) is used in the precursor solution. Secondly, a relatively low content of sulfur source (such as 1:2 or 1:3) in the precursor solution resulted in a heterogeneous composition of the layer – a mixture of phases as well as a visually inhomogeneous coverage – and is thus undesired. Conversely, the abundance of sulfur in the precursor solution, such as 1:6 or 1:9, suppresses the formation of oxide during the growth of the Sb_2_S_3_ film at a substrate temperature of 250 °C in the CSP process in air.

We reported the first results on the use of spray-grown Sb_2_S_3_ in planar TiO_2_/Sb_2_S_3_/P3HT solar cells. Low-cost hybrid solar cells with crystalline Sb_2_S_3_ nanoparticulate absorber fabricated by chemical spray pyrolysis in air exhibit a conversion efficiency of 1.3% from an active area of 1 cm^2^. Further development is in progress, the outlook will be to grow the Sb_2_S_3_ layer onto a structured substrate such as a ZnO nanorod layer.

## Experimental

### Technology of layers and solar cells

We used a commercial 300 nm indium tin oxide (ITO) coated 1.1 mm glass with a sheet resistance of 10 Ω·sq.^−1^ from Zentrum für Sonnenenergie- und Wasserstoff-Forschung Baden-Württemberg (ZSW).

The TiO_2_ layer was deposited by ultrasonic CSP onto the ITO using a spray solution of titanium(IV) isopropoxide precursor (0.1 mol·L^−1^) and acetylacetonate (both solutions from Merck Schudart OHG) at a molar ratio of 1:4 dissolved in ethanol [67–68], using a substrate temperature of 340 °C, followed by annealing at 450 °C for 30 min in air to assure the formation of the anatase phase. The thickness of the TiO_2_ layer remained between 80 and 100 nm as estimated from scanning electron microscopy (SEM) images.

For growing Sb_2_S_3_, the precursor solution was prepared inside a glove box with controlled humidity (less than 14 ppm). The solution contained SbCl_3_ (with Sb^3+^ concentration of 15 mmol/L) and SC(NH_2_)_2_ precursors at a molar ratio of 1:*x* (*x* = 2, 3, 6, 9) in methanol as the solvent. SbCl_3_ was purchased from Sigma-Aldrich (≥99.0%, p.a.) and SC(NH_2_)_2_ from Merck (≥99.0%, p.a.). The precursor solution was ultrasonically nebulized at 1.5 MHz and the resulting aerosol was guided onto the TiO_2_ substrate by using compressed air as the carrier gas at a flow rate of 5 L·min^−1^.

The deposition temperature 250 °C was chosen by relying on the fact that the SbCl_3_ and thiourea (tu) form the Sb(tu)_2_Cl_3_ complex compound in methanol and undergo thermal decomposition slightly above 200 °C according to a TG-DTA study [69]. This is characteristic for different metal chloride–thiourea complexes such as In(tu)_3_Cl_3_ [25,70], Sn(tu)_2_Cl_2_ [28] and CuCl–tu complexes with different stoichiometry [71]. To generalize, not only chlorides but also the SbI_3_ complex with tu, Sb(tu)_3_I_3_, decompose at around 200 °C [72]. Also, our preparatory study on the use of SbCl_3_ and tu for growing Sb_2_S_3_ films by spray pyrolysis [23] indicated the use of 250 °C as a suitable growth temperature according to thermal analysis of the Sb(tu)_2_Cl_3_ with tartaric acid as the complexing agent. Besides the requirement stemming from the use of the precursor, temperatures of above 225 °C are required for the crystallization of Sb_2_S_3_ [44].

Samples were prepared with the number of Sb_2_S_3_ growth cycles ranging from three up to 18. One deposition cycle accounts for a condition when the spray nozzle has passed twice over the substrate with an area of about 20 cm^2^ in 3 min, using 12 mL of solution. Thus, the solution consumption rate was around 0.2 mL·min^−1^·cm^−2^.

To apply the P3HT as the hole conductor, the glass/ITO/TiO_2_/Sb_2_S_3_ stack was immersed into a room temperature solution of 2 wt % regioregular poly(3-hexylthiophene-2,5-diyl), by Sigma-Aldrich, in chlorobenzene, followed by drying of the sample at 50 °C for 10 min in air and further drying of the sample in vacuum (4·10^−6^ Torr) at 170 °C for 5 min. The thickness of P3HT layer remained below 400 nm, as estimated from SEM images.

The Au layer was deposited onto the P3HT by thermal evaporation of metallic Au for 10 min under a pressure of 2·10^−6^ Torr through a metal mask with a number of holes that had an area of 1.7 mm^2^ each. Alternatively, larger contacts of 1 cm^2^ were physically isolated by scribing the solar cell. The thickness of the Au layer was around 50 nm.

For light soaking of the glass/ITO/TiO_2_/Sb_2_S_3_/P3HT/Au solar cell from the glass side we used a 300 W quartz–tungsten–halogen (QTH) lamp at a distance that resulted in 100 mW·cm^−2^ irradiance on the solar cell. The light soaking was carried out under open-circuit conditions for a minimum of 20 min, in a stream of ambient air provided by a cooling fan, until maximum conversion efficiency was reached.

### Characterization of layers and solar cells

For structural characterization, Raman spectra were measured in a backscattering configuration at room temperature using a confocal micro-Raman spectrometer HORIBA Jobin Yvon Model HR 800. The excitation radiation wavelength was 532 nm, the power density was sufficiently low without excessive heating the sample area of ca 100 μm^2^, during 100 s of data acquisition there were no signs of thermal alteration of the sample.

The analysis of the elemental composition of the films was performed by energy dispersive X-ray (EDX) analysis using Bruker spectrometer with ESPRIT 1.8 system at the Zeiss HR FESEM Ultra 55 scanning electron microscope (SEM) operating at an accelerating voltage of 10 kV. The same SEM system was used for visualization of the morphology of the layers and of the cross-section of the solar cells at an electron beam accelerating voltage of 4 kV.

Current–voltage scans of the solar cells were used to obtain the principal characteristics of the solar cells: voltage under open circuit conditions (*V*_OC_), current density under short-circuit conditions (*J*_SC_), the fill factor (FF) and the conversion efficiency (η) under AM1.5 standard conditions. The standard conditions were simulated by using the illumination of a 300 W quartz–tungsten–halogen (QTH) lamp at a distance that was adjusted by using a calibrated silicon solar cell as the detector. The active area of the solar cells is defined by the back contact area of 1.7 mm^2^ or 1 cm^2^.

The total transmittance spectra of the layers and solar cells were measured in the wavelength range of 300–1500 nm on a Jasco V-670 spectrophotometer equipped with an integrating sphere. The absorption coefficient was calculated as α = d^−1^·ln(*T*^−1^), where *d* is the layer thickness and *T* is the total transmittance, i.e. the sum of diffuse and specular transmittance. A Tauc plot was used to determine the bandgap of Sb_2_S_3_ layers assuming a direct optical transition.

The external quantum efficiency (EQE) of the solar cells was measured in the range of 350–1000 nm on a Newport Oriel kit that contains a 300 W Xe lamp, high-resolution monochromator (Cornerstone 260), digital dual-channel lock-in detector (Merlin), and a calibrated silicon reference detector. The Xe lamp is a light source that simulates the conventional AM1.5 spectrum for testing solar cells. The dispersed light from the Xe lamp (incident on the solar cell as monochromatic light) was optically chopped at 30 Hz. The EQE is defined as the number of collected charge carriers per incident photon. The EQE is a unitless characteristic (EQE < 1) given by EQE(λ) = (*hc*/*q*λ) × *J*_SC_(λ)/*P*(λ), where *J*_SC_(λ) (A·m^−2^) is the spectrally resolved short-circuit current of the solar cell, *P*(λ) (W·m^−2^) is the calibrated light intensity incident on the solar cell as function of wavelength λ, and *hc*/*q*λ is the energy (eV) of the incident photon. The samples were covered with a black cloth during the EQE measurements to avoid any photoactive effects in the component layers due to the ambient white light, only the low-intensity monochromatic light was incident on the cell. To validate the EQE result, the integrated product the EQE(λ) and solar irradiance *I*_AM1.5_(λ) was calculated by using the online tool Open Photovoltaics Analysis Platform, and compared with the *J*_SC_ obtained from the current–voltage scan.

## Supporting Information

Additional SEM images, optical transmittance spectra, and current–voltage curves.

File 1 Additional experimental data.
