# Supplementary material for: Sb2S3 grown by ultrasonic spray pyrolysis and its application in a hybrid solar cell
Source: Beilstein J Nanotechnol. 2016 Nov 10;7:1662–73. doi: 10.3762/bjnano.7.158 (PMC5238638; doi:10.3762/bjnano.7.158)
Supplement: File 1 — Additional experimental data. [file Beilstein_J_Nanotechnol-07-1662-s001.pdf]

Supporting Information

for

Sb_2S_3 grown by ultrasonic spray pyrolysis and its application in a hybrid solar cell

Erki Kärber[1]*, Atanas Katerski[1], Ilona Oja Acik[1], Arvo Mere[1], Valdek Mikli[2] and Malle Krunks[1]

Address: [1]Laboratory of Thin Film Chemical Technologies, Department of Materials Science, Tallinn University of Technology, Ehitajate tee 5, 19086 Tallinn, Estonia and [2]Chair of Semiconductor Materials Technology, Department of Materials Science, Tallinn University of Technology, Ehitajate tee 5, 19086 Tallinn, Estonia

Email: Erki Kärber* - erki.karber@ttu.ee

* Corresponding author

Additional experimental data

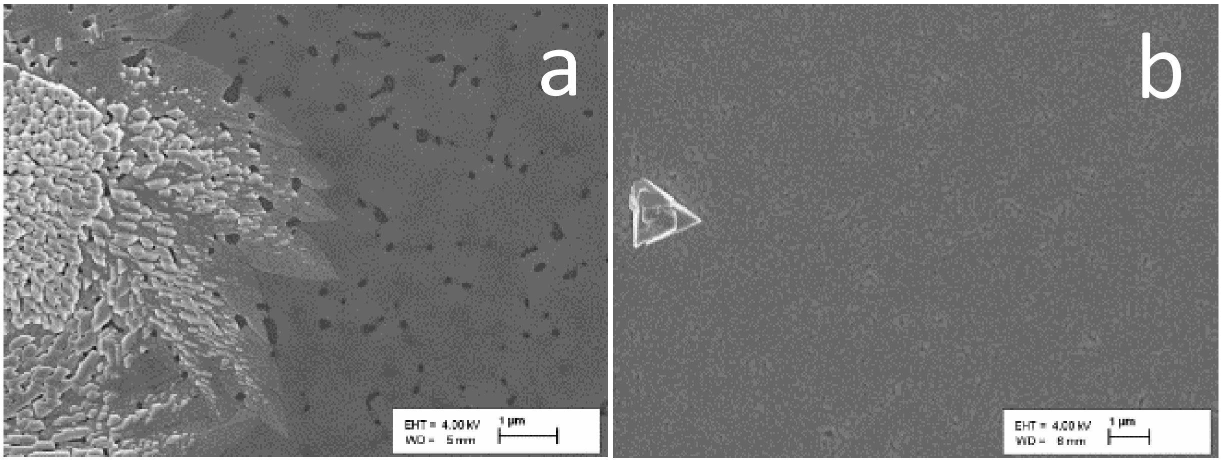

Figure S1: Top-view SEM images of Sb_2S_3 layers grown by ultrasonic-CSP using a) a solution with Sb/S source ratio of 1:3 and b) a solution with Sb/S source ratio of 1:2. Crystalline and amorphous parts are evident in image a. The single tetrahedral crystal in image b is composed of Sb and O according to EDX.

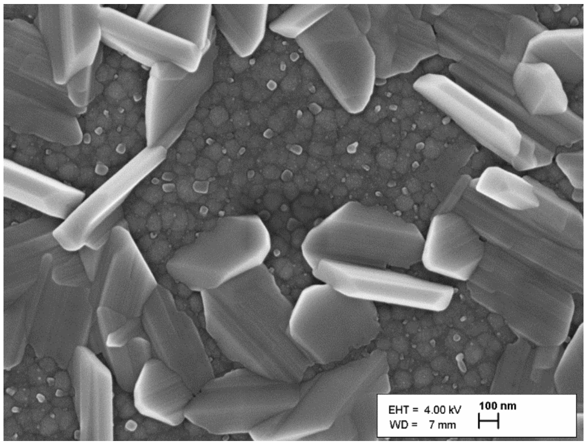

Figure S2: Top-view SEM image of Sb_2S_3 crystals grown by 7-cycles of spraying of solutions with Sb/S precursor ratio of 1:6 onto a TiO_2 substrate kept at 250 °C.

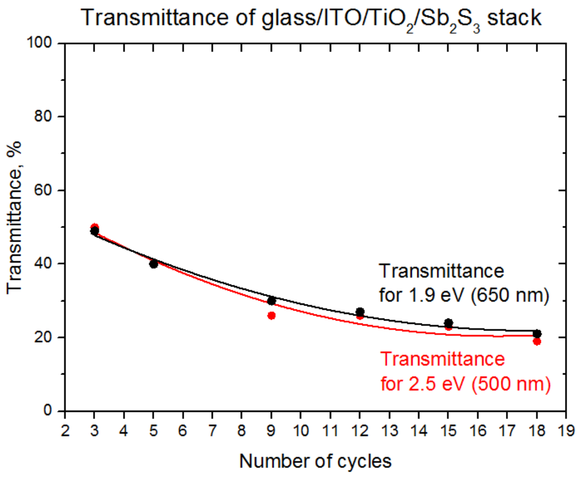

Figure S3: Optical transmittance of the glass/ITO/TiO_2/Sb_2S_3 stack at two different photon energies (1.9 eV and 2.5 eV) as a function of Sb_2S_3 growth cycles. The transmittance includes the specular and the diffuse component.

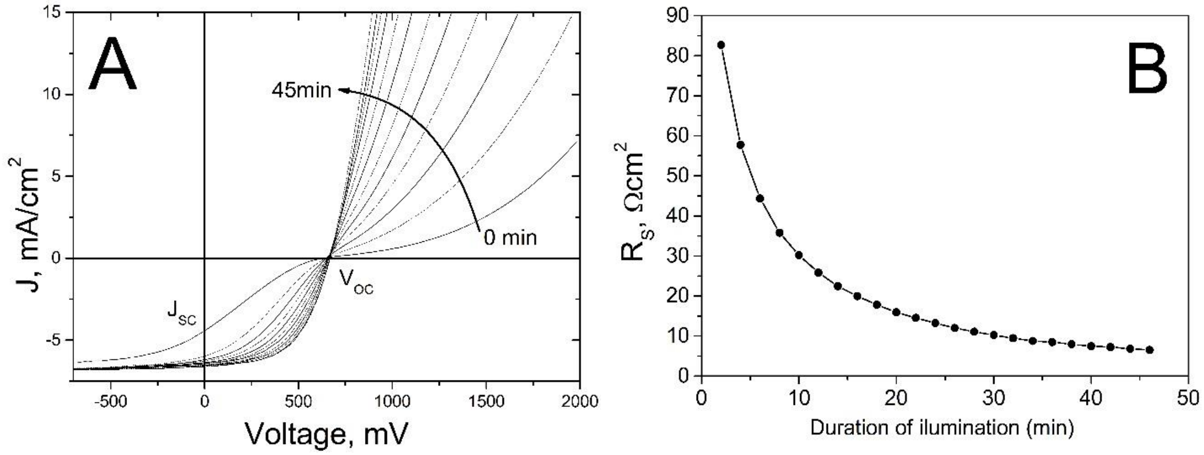

Figure S4: Current–voltage characteristics (A) and series resistance (B) of the glass/ITO/TiO_2/Sb_2S_3/P3HT/Au solar cells as a function of the duration of light-soaking using A.M1.5 illumination up to 45 min. The series resistance of the solar cell is calculated using the slope of the *I*–*V* curve rightwards from the location of the V_{OC}.
